# P-2054. When Crisis Meets Context: Social Determinants of Health for People Living with HIV Presenting to an Urban Emergency Department

**DOI:** 10.1093/ofid/ofaf695.2218

**Published:** 2026-01-11

**Authors:** Amanda Varnauskas, Gaby Dashler, David Rudolph, Holly Everett, Sarah Hill-Yeterian, Bhakti Hansoti

**Affiliations:** Johns Hopkins University, Baltimore, MD; Johns Hopkins School of Medicine, Baltimore, Maryland; Johns Hopkins University School of Medicine, Baltimore, Maryland; Johns Hopkins University, Baltimore, MD; Johns Hopkins University, Baltimore, MD; Johns Hopkins University, Baltimore, MD

## Abstract

**Background:**

Social determinants of health (SDOH) are non-clinical factors that influence health outcomes, and Emergency Departments (ED) see a high volume of patients disproportionately affected by social vulnerabilities. A large proportion of people living with HIV (PLWH) frequent the ED, and we suspect that quantifying SDOH among PLWH presenting to the ED will help us better understand their service delivery needs.

**Figure:**
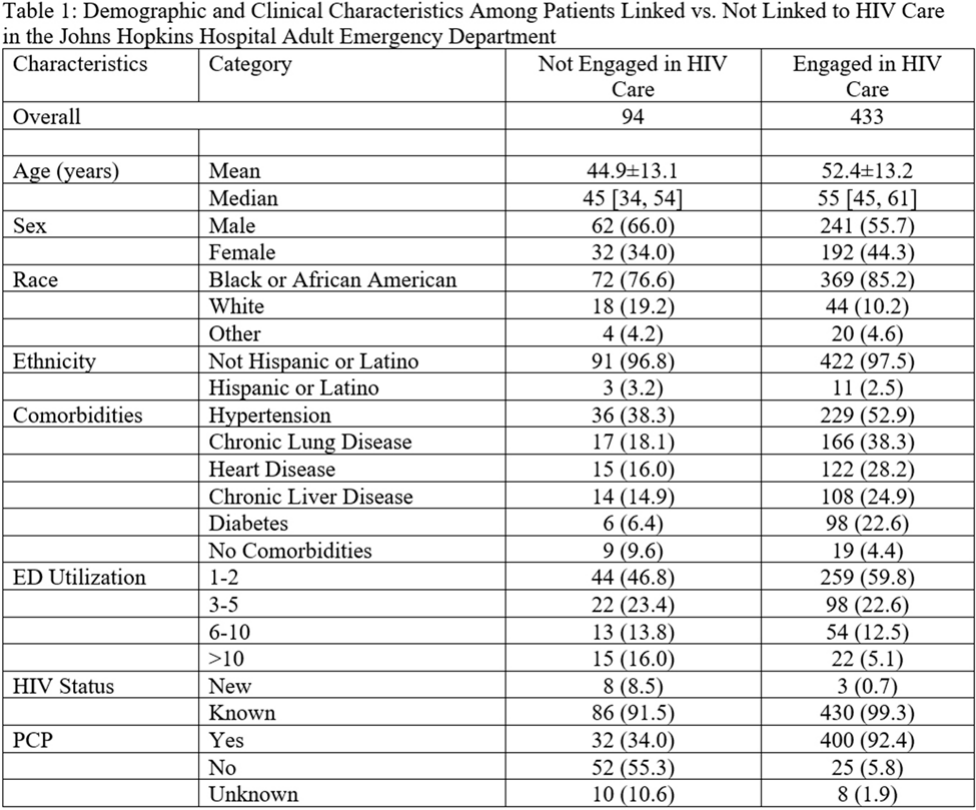

**Figure:**
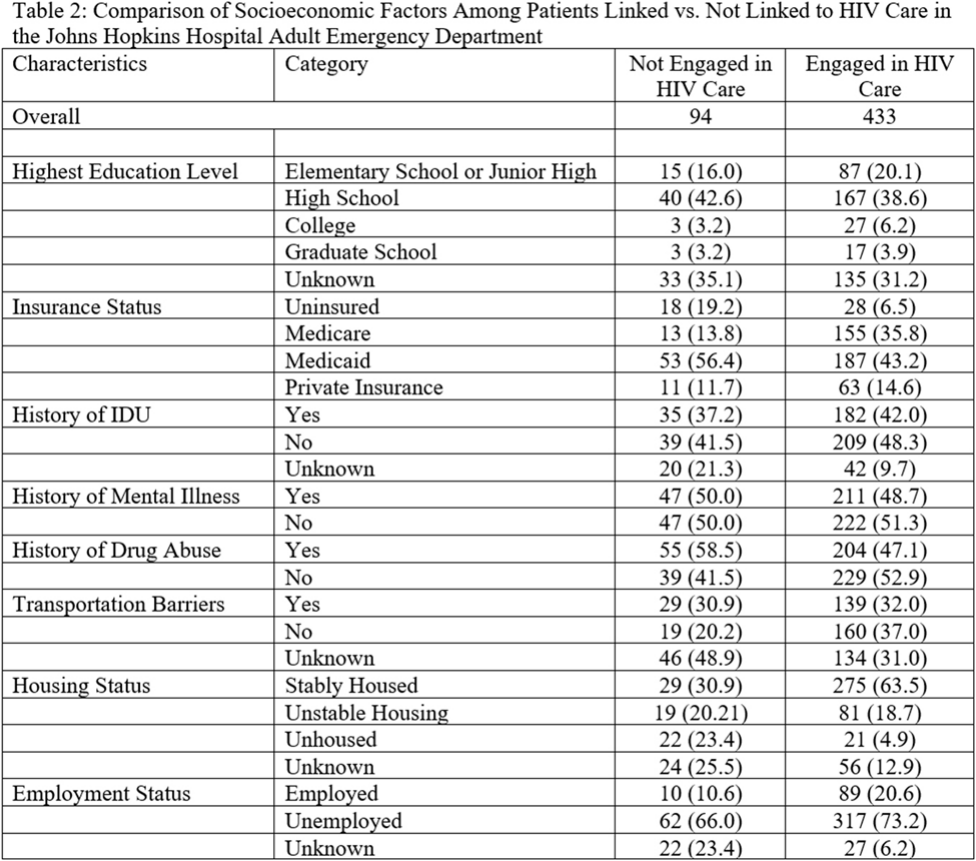

**Methods:**

Our team conducted a retrospective chart review of all patients with an HIV diagnosis (known or new) presenting to an urban ED between 01/01/2020 and 12/31/2020. We abstracted data on demographics, ED encounter information, if linked to care, defined as a documented HIV care appointment in the last 6 months, and various SDOH (education, employment, transportation, housing stability, food security, substance use), which were recorded in REDCap. Data were analyzed using simple descriptive statistics and multivariable logistic regression to evaluate associations between SDOH and the primary outcome of HIV linkage to care.

**Figure:**
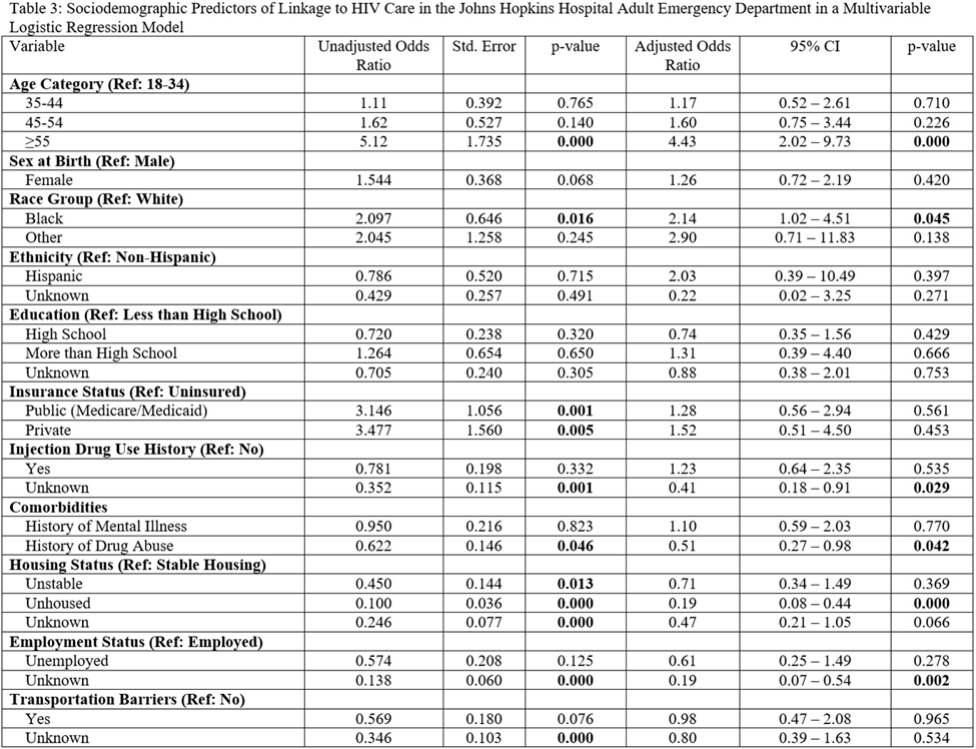

**Results:**

In total, 527 unique patients were included in our cohort, of which 303 (57.5%) were male, the median age was 53 years (IQR 41-60), and 441 (84%) identified as Black. The majority (n=433, 82%) were linked to HIV care. Our cohort had a high prevalence of social vulnerabilities namely documented history of drug use (n=259, 49%), unemployment (n=379, 72%), lack of stable housing (n=143, 27%), and lack of reliable transportation (n=168, 32%), while few were uninsured (n=46, 0.09%). Being unhoused (aOR: 0.19, 95% CI: 0.08 – 0.44) and history of drug use (aOR: 0.51, 95% CI: 0.27 – 0.98) were significantly associated with lower odds of linkage to care. Additionally, SDOH were inconsistently documented in patient files, with variable-missingness ranging from 9.3-34%.

**Conclusion:**

As anticipated, patients with a lack of housing and a history of substance use have a significant risk of not linking to care, which could impact their HIV treatment engagement. This underscores the need to advocate for ED-based consults for social work and case management, with referral to HIV programs. Furthermore, inconsistent documentation of SDOH compromises our understanding of this population and mitigates our ability to provide appropriate and contextualized support services.

**Disclosures:**

All Authors: No reported disclosures

